# Prenatal Molecular Diagnosis of COL2A1-Associated Stickler Syndrome: Genotype–Phenotype Correlation in a Resource-Limited Healthcare Setting

**DOI:** 10.3390/ijms27052227

**Published:** 2026-02-27

**Authors:** Elitsa Gyokova, Eleonora Hristova-Atanasova, Zlatko Kirovakov, Kamelia Dimitrova

**Affiliations:** 1 Department of Obstetrics and Gynecology, Faculty of Medicine, Medical University-Pleven, 5800 Pleven, Bulgaria; elitca.gaokova@mu-pleven.bg; 2 University Hospital “Saint Marina”-Pleven, 5800 Pleven, Bulgaria; 3 Department of Social Medicine and Public Health, Faculty of Public Health, Medical University of Plovdiv, 4002 Plovdiv, Bulgaria; 4 Faculty of Public Health and Health Care, Burgas State University “Prof. d-r Asen Zlatarov”, 8000 Burgas, Bulgaria; 5 Faculty of Medicine, Medical University-Pleven, 5800 Pleven, Bulgaria; k.kamenova.96@gmail.com

**Keywords:** Stickler syndrome, *COL2A1*, frameshift variant, prenatal molecular diagnosis, monogenic connective tissue disorder, genotype–phenotype correlation

## Abstract

Stickler syndrome is a monogenic connective tissue disorder primarily caused by pathogenic variants in collagen-related genes, most commonly *COL2A1*. Prenatal diagnosis remains challenging, particularly in healthcare systems with limited access to molecular genetic testing. We report a prenatal case of suspected craniofacial anomaly detected on second-trimester ultrasound. Fetal DNA obtained by amniocentesis underwent next-generation sequencing. Parental testing was performed to assess inheritance. It was confirmed that autosomal dominant Stickler syndrome type I (ORPHA:90653) was caused by a heterozygous pathogenic frameshift variant in *COL2A1* (c.3137del) that was inherited from the mother and identified in the fetus. Micrognathia was identified during prenatal ultrasound, and postnatal evaluation revealed characteristics that were consistent with Pierre Robin sequence and connective tissue involvement. The molecular discoveries elucidated the observed phenotype and facilitated multidisciplinary perinatal management. This case underscores the indispensable function of molecular diagnostics in the prenatal identification of monogenic disorders, including Stickler syndrome, in cases where conventional karyotyping is inadequate. Targeted clinical surveillance and family counseling are facilitated by early genetic confirmation. The report also emphasizes the necessity of incorporating molecular diagnostics into routine prenatal care for rare genetic diseases and the systemic limitations in access to genomic testing. Although the identified variant has been previously reported, this case highlights the clinical and diagnostic value of prenatal molecular confirmation in a resource-limited healthcare setting.

## 1. Introduction

DNA, RNA, chromosomes, proteins, or metabolites are analyzed to identify heritable disease-related genotypes, mutations, phenotypes, or karyotypes for clinical purposes. This process is known as genetic examination. This is essential for the diagnosis of monogenic disorders, carrier detection, disease risk prediction, and precision clinical management, and it encompasses a wide variety of techniques, such as targeted gene sequencing, whole-exome sequencing (ES), whole-genome sequencing (GS), and cytogenetic methods [1,2].

The diagnostic yield for rare inherited diseases has been significantly enhanced by the simultaneous interrogation of multiple disease-associated genes at single-base resolution, as demonstrated in large clinical studies reported in the literature [3,4], primarily due to advancements in next-generation sequencing (NGS) technologies, particularly ES and GS.

Pathogenic variants are identified in approximately 25–52% of patients with suspected rare genetic disorders by NGS-based approaches, which significantly outperform conventional cytogenetic techniques in diagnostic sensitivity, particularly when trio sequencing is employed.

Karyotyping is a specific type of genetic examination involving microscopic evaluation of chromosome number and structure in metaphase cells and remains the gold standard for detecting aneuploidies and large chromosomal rearrangements. It is commonly employed in the evaluation of congenital anomalies, recurrent pregnancy loss, and hematologic malignancies; however, it is fundamentally restricted to the detection of macroscopically visible abnormalities [5,6].

Genetic examination therefore represents a broad category of laboratory techniques, whereas karyotyping specifically targets large-scale chromosomal alterations and lacks the resolution necessary to identify pathogenic single-gene variants [1,5,6].

In monogenic connective tissue disorders, the distinction between conventional cytogenetic approaches and molecular sequencing-based methods is particularly important, as the causative mutations are typically submicroscopic—such as single-nucleotide variants, small insertions/deletions, or splice-site mutations—and are therefore imperceptible to karyotyping or chromosomal microarray analysis [7,8].

Conversely, targeted gene panels, ES, and GS facilitate the direct interrogation of disease-associated genes that encode extracellular matrix components, such as collagen genes, thereby significantly increasing diagnostic sensitivity and facilitating precise molecular classification.

Stickler syndrome is a hereditary connective tissue disorder that is distinguished by a variety of ocular abnormalities, including congenital high myopia and vitreoretinal degeneration, which are associated with a high risk of retinal detachment. Additionally, the disorder is characterized by sensorineural hearing loss; distinctive craniofacial features, such as midface hypoplasia, micrognathia, cleft palate, or bifid uvula; and early-onset joint hypermobility and osteoarthritis [6,9,10,11]. The most common mode of inheritance is autosomal dominant, as a result of pathogenic variants in *COL2A1*, *COL11A1*, and *COL11A2*. Conversely, the rarer autosomal recessive forms involve *COL9A1*, *COL9A2*, or *COL9A3* [6,9,10,11].

The type II collagenopathy spectrum, which includes Stickler syndrome type I (OMIM #108300), spondyloepiphyseal dysplasia congenita, and Kniest dysplasia, is primarily caused by mutations in *COL2A1* [12].

Stickler syndrome type I and ocular-predominant phenotypes are most frequently associated with haploinsufficiency resulting from nonsense or frameshift variants in *COL2A1*. Missense mutations that affect the collagen triple-helix domain frequently produce more severe skeletal and craniofacial manifestations, reflecting substantial genotype–phenotype heterogeneity [11,13].

Prenatal diagnosis of Stickler syndrome is feasible when a pathogenic variant has been identified in a parent. According to published clinical literature, targeted molecular testing may be performed on fetal DNA obtained via chorionic villus sampling or amniocentesis, and preimplantation genetic testing is an additional option [6]. In the absence of a known familial variant, ultrasound may reveal suggestive but nonspecific findings, such as micrognathia or the Pierre Robin sequence, although normal imaging does not exclude the diagnosis [6,14,15,16].

Karyotyping is not diagnostic for Stickler syndrome because the pathogenic variants are submicroscopic and necessitate sequencing-based molecular methods for identification [6].

Notwithstanding the international recommendations from the American College of Medical Genetics and Genomics (ACMG) advocating for molecular testing of suspected type II collagen disorders, access to NGS-based diagnostics is markedly inconsistent across healthcare systems due to disparities in infrastructure, reimbursement, and specialist availability [17].

In Bulgaria, karyotyping is presently the sole genetic test routinely reimbursed by national health insurance, making prenatal molecular diagnosis of Stickler syndrome predominantly unattainable. Due to the inability of cytogenetic methods to identify pathogenic variants in collagen genes, prenatal assessment is limited to ultrasound results and familial history, lacking conclusive genetic verification [6,18]. This constraint hinders genetic counseling, recurrence risk evaluation, and informed reproductive choices [6,16,19].

This diagnostic gap illustrates the overarching difficulty of incorporating genomic medicine into standard prenatal care for rare monogenic disorders, resulting in delayed diagnoses, diminished clinical readiness, and ongoing health disparities, as emphasized in recent studies on genomic medicine implementation [20,21]. Although the pathogenic variant identified in this case has been previously reported, the present study focuses on the clinical and diagnostic pathway leading to prenatal molecular confirmation, the integration of fetal imaging with parental phenotyping, and the implications of genomic diagnosis in a healthcare system with limited access to sequencing-based testing.

## 2. Materials and Methods

### 2.1. Ethical Considerations and Informed Consent

The prenatal diagnostic procedure was conducted in accordance with the Declaration of Helsinki. Clinical genetic counseling was provided to the parents prior to any intervention. Written informed consent was obtained from the parents for the performance of invasive prenatal diagnosis, molecular genetic testing, and the subsequent publication of this case report, including all clinical data and imaging. The study was approved by the Ethics Committee of the Medical University–Pleven (approval number: P16/2025).

### 2.2. Prenatal Imaging and Clinical Evaluation

High-resolution ultrasound was employed to evaluate the fetal anatomy. A comprehensive second-trimester anomaly scan was performed at 21 weeks and 2 days of gestation, following a standard first-trimester screening at 12 weeks and 0 days of gestation. To assess the sagittal fetal facial profile and identify craniofacial markers, a GE Voluson E8 ultrasound system was implemented. The Fetal Medicine Unit, University Hospital “Saint Marina,” Pleven, Bulgaria, conducted all assessments.

### 2.3. DNA Isolation and Sample Acquisition

At 23 weeks of gestation, ultrasound-guided amniocentesis was employed to obtain fetal DNA. To facilitate a trio-based sequencing method and determine the inheritance pattern of any detected variants, peripheral venous blood samples were simultaneously collected from both parents. Genomic DNA was extracted from parental blood specimens and uncultured amniocytes. Prior to sequencing, Qubit fluorometric quantification was implemented to assess the quantity and quality of DNA.

### 2.4. Bioinformatics Processing and Genetic Analysis

An average coverage of approximately 30× was achieved by HaploX (Hong Kong) with an Illumina NovaSeq 6000 platform for next-generation sequencing (NGS). The GRCh38/hg38 human reference genome was used to align the raw sequencing data. Standard clinical bioinformatics pipelines were employed to execute variant calling and annotation, which included the filtration of infrequent, protein-altering variants in genes associated with disease.

### 2.5. Interpretation and Variant Nomenclature

The Human Genome Variation Society (HGVS) nomenclature was used to characterize the identified variant in the *COL2A1* gene (c.3137del). Variant pathogenicity was assessed in accordance with the standards established by the ACMG. The clinical classification was verified by ClinVar and curated variant databases, while population allele frequencies were assessed using gnomAD. A trio-based analysis was employed to assess segregation, thereby validating maternal inheritance.

### 2.6. Data Accessibility

Sequencing data that verify the findings of this study can be obtained from the corresponding author upon reasonable request.

## 3. Results

The aim of this case report is to present a neonate with molecularly confirmed Stickler syndrome type 1 (ORPHA:90653; OMIM:108300) and to emphasize the roles of prenatal ultrasound suspicion, sequencing-based genetic diagnosis, and multidisciplinary perinatal management and the clinical impact of limited access to molecular testing.

A 27-year-old pregnant woman was managed at the Fetal Medicine Unit at University Hospital “Saint Marina,” Pleven, Bulgaria, for routine first-trimester screening. The maternal medical history was unremarkable, and no known hereditary disorders were reported. There was no reported consanguinity or family history of congenital anomalies. A non-invasive prenatal test performed at 10 + 1 gestational weeks indicated low risk for common chromosomal aneuploidies in a male fetus. First-trimester ultrasound at 12 + 0 weeks demonstrated normal fetal anatomy, and combined screening revealed low risk for major chromosomal abnormalities.

**Figure 1:**
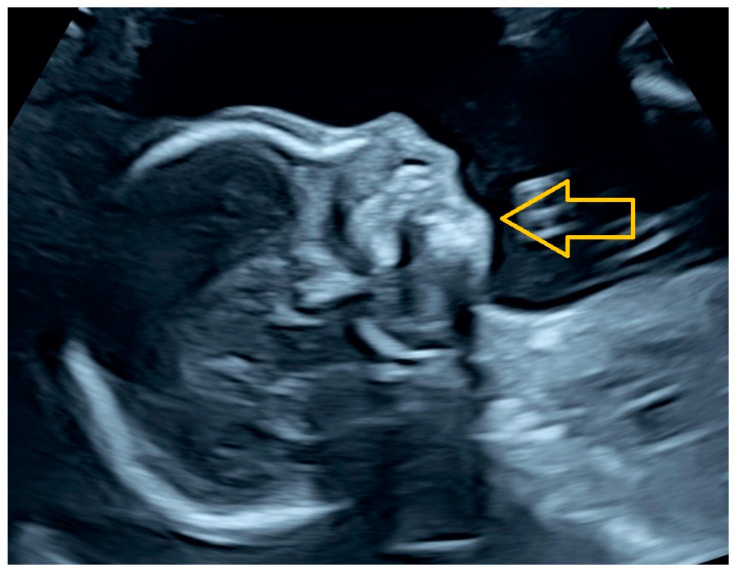
Results of a prenatal ultrasound: Marked micrognathia is evident in the sagittal fetal facial profile at 21 + 2 weeks of gestation, which is indicative of craniofacial involvement associated with monogenic connective tissue disorders. The arrow indicates the region of mandibular hypoplasia consistent with micrognathia.

During routine second-trimester evaluation at 21 + 2 weeks of gestation, fetal growth parameters were within normal limits; however, pronounced micrognathia was identified on sagittal facial profile ultrasound (Figure 1). No additional structural anomalies were detected. Mild maternal micrognathia was also observed on clinical examination, raising suspicion of a possible inherited craniofacial phenotype. Following the genetic confirmation, a retrospective clinical evaluation of the mother revealed that she had previously experienced mild manifestations consistent with the syndrome, including unspecified myalgia/arthralgia and ophthalmalgia, which had not been formally investigated prior to the fetal diagnosis. Based on these findings, a monogenic connective tissue disorder was considered, and genetic counseling was arranged.

An uncomplicated amniocentesis was conducted at 23 weeks of gestation after rigorous counseling.

A genome-based approach was employed to conduct NGS on fetal DNA, which was subsequently followed by parental testing. Stickler syndrome type I (ORPHA:90653) was confirmed by the identification of a heterozygous pathogenic frameshift variant in the *COL2A1* gene (c.3137del) in the fetus. The variant was classified as pathogenic according to ACMG-ClinGen criteria and is expected to lead to the haploinsufficiency-induced loss of function of type II collagen. The mother’s mild clinical phenotype was correlated with the detection of the same variant. No additional pathogenic variants that were clinically relevant were identified.

**Figure 2:**
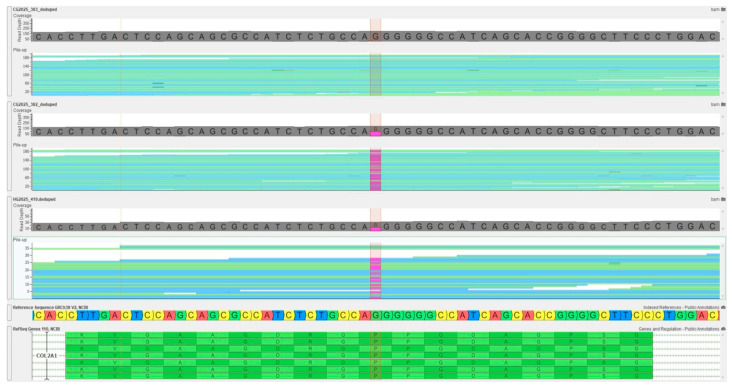
Molecular confirmation of *COL2A1* frameshift variant. An identical variant was detected in the maternal sample, which is consistent with autosomal dominant inheritance. The *COL2A1* gene in fetal DNA has a heterozygous deletion, c.3137del, as demonstrated by a next-generation sequencing read alignment.

The alignment of sequencing reads revealed a consistent heterozygous deletion at the affected locus in both fetal and maternal samples (Figure 2).

Given the ultrasound diagnosis of solitary micrognathia, conventional cytogenetic testing was explored as part of the differential diagnostic work-up, specifically to rule out chromosomal aneuploidies or structural rearrangements linked to craniofacial deformities. However, in the absence of additional structural abnormalities and given the maternal craniofacial phenotype suggestive of a heritable connective tissue disorder, sequencing-based molecular analysis was prioritized to detect a potential monogenic etiology that would be undetectable by standard karyotyping.

A personalized perinatal care plan was developed by a multidisciplinary team that included fetal medicine specialists, clinical geneticists, neonatologists, otorhinolaryngologists, orthopedic specialists, and surgical consultants as soon as the molecular diagnosis was confirmed.

Genetic counseling addressed inheritance risk, anticipated clinical course, and long-term surveillance strategies.

At 38 weeks of gestation, a spontaneous vaginal delivery occurred at the University Hospital “Saint Marina” in Pleven, Bulgaria. The neonate’s birth weight was 3100 g, and its length was 48 cm. Its head circumference was 34 cm. The Apgar scores were 8 at one minute and 10 at five minutes. Distinct dysmorphic characteristics, including a cleft palate, a short lingual frenulum, a flat forehead, downslanting palpebral fissures, significant micrognathia, and microretrognathia, were identified during the postnatal examination, which indicates a Pierre Robin sequence. Additional observations indicated that the toes were wide, with underdeveloped nails, and there was a clinical suspicion of joint hypermobility.

On the second day of life, the molecular diagnosis was verified through genetic consultation. Annual ophthalmologic surveillance, audiologic evaluation, and orthopedic evaluation were recommended as post-diagnostic measures.

Due to feeding difficulties associated with craniofacial anomalies, the neonate required enteral nutrition via orogastric tube for 8 weeks during the initial hospitalization period. Transfontanelle ultrasound examination demonstrated normal brain anatomy, and microbiological studies were normal.

The initial newborn hearing screening revealed bilateral abnormalities; however, the results were reconciled to normal during a subsequent examination at 12 weeks of age. Growth parameters at discharge were normal for the newborn charts. Postnatally, the infant has been followed by an ophthalmologist in an outpatient setting; however, detailed clinical findings from these examinations were not formally recorded in the available hospital medical records at the time of this report.

During four months of follow-up, the infant demonstrated physiological neurodevelopmental progression and successful oral feeding without tube support. Corrective palatal surgery is planned at approximately 10 months of age.

## 4. Discussion

Stickler syndrome is a hereditary connective tissue disorder characterized by variable combinations of ocular, craniofacial, auditory, and musculoskeletal abnormalities. The most common clinical features include high myopia, vitreous anomalies, and a markedly increased risk of retinal detachment; craniofacial findings such as midface hypoplasia, micrognathia, and cleft palate; sensorineural hearing loss; and early-onset joint hypermobility and osteoarthritis. Skeletal manifestations may include epiphyseal dysplasia and spinal abnormalities such as scoliosis or platyspondyly.

The severity and combination of features that affect individuals vary significantly [6,10,11,22,23]. This clinical heterogeneity is particularly relevant in prenatal settings, where manifestations may be incomplete or subtle, complicating phenotype-based recognition. In the present case, variability was evident in the discordance between the mild maternal phenotype and the more pronounced neonatal craniofacial involvement.

This variability is indicative of both genetic heterogeneity and mutation-specific molecular mechanisms that affect collagen biosynthesis and extracellular matrix integrity, and type II collagenopathy summaries [6,12]. Such intrafamilial variability has important implications for prenatal risk assessment and counseling, as parental manifestations may be minimal despite transmission of a pathogenic variant.

The most prevalent genetic causes are autosomal dominant mutations in collagen genes, specifically *COL2A1* (type I Stickler syndrome), *COL11A1*, and *COL11A2*. Rare autosomal recessive forms are the result of biallelic mutations in *COL9A1*, *COL9A2*, or *COL9A3*, and they may be distinguished by more severe ocular findings and hearing loss [9,18,24,25,26]. Hearing loss and joint disease are more frequently associated with *COL11A1/2* and *COL9A1/2/3* mutations, while ocular findings are most frequently associated with *COL2A1* mutations. The specific gene involved can affect the phenotype [6]. Large cohort genotype–phenotype analyses, including the 100-patient series by Hoornaert et al. [11], support this gene-dependent phenotypic pattern and further underscore marked intrafamilial variability.

Importantly, the pathogenic *COL2A1* variant identified in this case has been previously reported in clinical variant databases and published genotype–phenotype cohorts, including the series described by Hoornaert et al. [11]. Therefore, the contribution of the present report does not lie in the identification of a novel molecular finding, but in the clinical documentation of prenatal diagnostic reasoning, phenotype–genotype integration, and management implications in a real-world healthcare setting with restricted access to genomic testing.

Diagnostic methods rely on clinical evaluation supported by molecular genetic testing. In the present case, clinical suspicion was triggered by prenatal ultrasound findings and strengthened by recognition of subtle maternal craniofacial characteristics, which together prompted targeted molecular investigation. This illustrates the diagnostic value of integrating fetal imaging with parental phenotyping when evaluating suspected inherited connective tissue disorders.

This case therefore highlights how previously characterized pathogenic variants may still present substantial diagnostic and management challenges depending on the timing of detection, clinical presentation, and healthcare infrastructure in which prenatal evaluation is performed.

The diagnosis is confirmed by using targeted gene panels or next-generation sequencing to identify pathogenic variants in the relevant collagen genes. Karyotyping is not diagnostic, as the causative mutations are submicroscopic. In families with a known mutation, molecular testing is essential for the definitive diagnosis, genetic counseling, and prenatal or preimplantation genetic diagnosis [6,11,25,26]. In addition to confirming diagnosis, molecular testing has the potential to reduce irreversible morbidity by enabling genotype-informed risk stratification, cascade testing in relatives, and earlier deployment of surveillance strategies. In this case, molecular confirmation directly influenced perinatal management, enabling anticipatory planning for airway support, feeding difficulties, and early specialist follow-up. This demonstrates that prenatal molecular diagnosis functions not only as a diagnostic tool but also as a management-directing intervention.

The *COL2A1* gene (Collagen Type II Alpha 1 Chain, OMIM:120140) encodes the alpha-1 chain, which is the primary component of type II collagen. Type II collagen is crucial for the normal development of the inner ear, eyes, and joints, as it provides structure and strength to connective tissues [6]. A broad range of autosomal dominant collagenopathies, which primarily affect the skeleton, joints, eyes, and occasionally the inner ear, are associated with pathogenic heterozygous variants in *COL2A1*. Although missense mutations in *COL2A1* are linked to more severe forms of skeletal dysplasias, loss-of-function variants have been primarily described in patients with milder phenotypes, such as Stickler syndrome type I [27]. The clinical picture observed and the diagnosis of Stickler syndrome type I are elucidated by the heterozygous state of the examined fetus, which carries a known pathogenic *COL2A1* variant (c.3137del) inherited from the mother. The probability of inheritance in the offspring of heterozygotes is 50%, irrespective of their gender. The observed phenotype is consistent with a loss-of-function mechanism, which aligns with the expected clinical spectrum associated with haploinsufficiency of type II collagen.

On a mechanistic level, frameshift variants like *COL2A1* c.3137del typically introduce premature termination codons, which lead to nonsense-mediated mRNA decay and reduced production of functional type II collagen (haploinsufficiency) rather than the synthesis of an abnormal dominant-negative protein [6,12]. This haploinsufficiency mechanism is consistent with the Stickler syndrome type I spectrum and explains the predominance of ocular and craniofacial involvement with comparatively milder skeletal disease [11]. Conversely, missense variants, particularly glycine substitutions in the collagen triple-helix domain, are frequently linked to more severe skeletal dysplasias and result in dominant-negative effects, as delineated in mutation [13,28].

The limitations of karyotyping and the necessity for molecular genetic testing to detect causative mutations are the primary challenges associated with the prenatal diagnosis of Stickler syndrome. Because these are single-gene mutations (primarily in *COL2A1*, *COL11A1*, *COL11A2*, *COL9A1*, *COL9A2*, or *COL9A3*) that are submicroscopic and not visible at the chromosomal level, karyotyping is unable to detect the pathogenic variants responsible for Stickler syndrome. Therefore, karyotyping is not a diagnostic tool for Stickler syndrome and will miss cases unless there is a rare, large chromosomal rearrangement involving these genes, which is not the typical mechanism in Stickler syndrome [6,18]. Molecular genetic testing is required to identify causative mutations. The diagnosis can only be established by detecting a pathogenic variant in one of the Stickler syndrome-associated genes. In the present case, isolated fetal micrognathia prompted consideration of both chromosomal and monogenic etiologies. Although conventional cytogenetic testing was considered, sequencing-based analysis was prioritized due to clinical suspicion of a single-gene disorder and the limited sensitivity of karyotyping for such conditions. This reflects real-world diagnostic reasoning in prenatal medicine.

For prenatal diagnosis, the procedure requires prior identification of the familial mutation in an affected parent. Once the familial mutation is known, targeted molecular testing can be performed on fetal DNA obtained by chorionic villus sampling or amniocentesis [6]. In the absence of a known familial mutation, prenatal diagnosis is not possible by molecular methods, and ultrasound findings (such as cleft palate or micrognathia) are nonspecific and insufficient for diagnosis. Furthermore, the American College of Obstetricians and Gynecologists states that single-gene disorders like Stickler syndrome require targeted molecular testing for prenatal diagnosis, and karyotyping is only useful for detecting chromosomal abnormalities, not single-gene disorders [18]. The inability of karyotyping to detect single-gene mutations and the requirement for prior identification of a familial pathogenic variant for molecular testing are the principal challenges in prenatal diagnosis [6,18].

Beyond the specific context of Stickler syndrome, multiple prenatal genomics cohorts demonstrate that sequencing-based methods substantially increase diagnostic yield in fetuses with ultrasound-detected structural anomalies, supporting the approach used in this case where imaging prompted molecular investigation. Specifically, a substantial number of fetuses with structural findings contain pathogenic single-gene variants that are unidentifiable by cytogenetic testing, as evidenced by large studies and exome sequencing cohorts [5,29]. This supports the idea that ultrasound is most effective when utilized as a trigger for sequencing, rather than as a standalone diagnostic tool. The present case provides a practical example of this diagnostic model.

Molecular genetic testing is not routinely available or covered by health insurance in Bulgaria, and karyotyping is covered but diagnostically inadequate. Karyotyping can only detect large chromosomal abnormalities and cannot identify the single-gene pathogenic variants in *COL2A1*, *COL11A1*, *COL11A2*, *COL9A1*, *COL9A2*, or *COL9A3* that cause Stickler syndrome. The diagnosis requires molecular genetic testing, either gene-targeted panels or comprehensive genomic sequencing, to detect these variants [6]. Patients and families in Bulgaria are unable to access definitive diagnostic tools necessary for accurate diagnosis, genetic counseling, and prenatal or preimplantation genetic testing since insurance coverage is restricted to karyotyping. This restriction obstructs cascade testing in at-risk relatives and prevents confirmation in suspected cases. Consequently, clinical management is contingent upon phenotype and family history, unless patients independently finance molecular testing [30,31]. This case therefore has translational relevance beyond a single family, illustrating how system-level constraints in genomic medicine implementation can directly impact diagnostic accuracy, timeliness of counseling, and clinical preparedness for monogenic disease, consistent with broader observations in genomic medicine for undiagnosed diseases [32] and large-scale rare disease genome sequencing evaluations [4]. In this instance, access to sequencing enabled definitive prenatal diagnosis and structured perinatal planning that would otherwise not have been possible. Accordingly, the clinical relevance of this report lies in illustrating a practical diagnostic pathway for integrating ultrasound findings, parental phenotyping, and targeted sequencing in healthcare systems where molecular genetic testing is not routinely accessible.

Early counseling is essential for families to comprehend the spectrum of clinical manifestations, recurrence risks, and inheritance patterns. This knowledge is essential for informed reproductive decision-making and preparation for potential neonatal complications, such as airway obstruction or feeding difficulties caused by craniofacial anomalies [6]. Psychological support is essential for families to navigate uncertainty, particularly when a definitive prenatal diagnosis is not feasible due to the absence of molecular testing. According to the American Society for Reproductive Medicine and the most recent literature on counseling models, counseling should address the emotional impact of risk, facilitate value-based decision-making, and provide support for coping strategies [33,34]. By acknowledging the limitations of the available testing and the potential for undetected complications, realistic expectations and reduced decisional regret are ensured. In the present case, molecular confirmation allowed individualized counseling based on a confirmed genetic etiology rather than probabilistic risk estimation.

To optimize outcomes for hearing, joint, and craniofacial issues, as well as to prevent irreversible complications, such as blindness from retinal detachment, postpartum follow-up planning is essential. This is because these complications can be prevented through the early identification and multidisciplinary management of affected neonates. The prophylactic vitreoretinal interventions and prevention-focused approaches have been linked to significant reductions in the risk of retinal detachment and blindness in Stickler syndrome [4,35,36]. Proactive ophthalmologic surveillance is particularly beneficial in this context. As a result, early molecular confirmation can be regarded as a gateway intervention that enables the timely referral of specialists, the provision of risk education, and the investigation of prophylactic strategies before the occurrence of irreversible complications.

Sequencing-based analysis was prioritized in this instance due to the clinical suspicion of a monogenic disorder and the known limited sensitivity of karyotyping for single-gene conditions, despite the fact that conventional cytogenetic testing was a consideration following the detection of fetal micrognathia.

The intrafamilial variability observed in this case—mild maternal manifestations in contrast to more pronounced fetal/neonatal craniofacial involvement—emphasizes the limitations of phenotype-based prediction in at-risk relatives and emphasizes the significance of molecular confirmation when prenatal findings raise suspicion for a monogenic connective tissue disorder. In the final analysis, molecular confirmation should be prioritized. Overall, this case illustrates how prenatal molecular diagnosis can refine clinical decision-making, improve perinatal preparedness, and enhance risk assessment in healthcare settings with limited access to genomic testing.

Although the molecular finding itself is not novel, the present case provides clinically relevant insight into the practical implementation of prenatal genomic diagnostics and demonstrates how early molecular confirmation can directly influence counseling, perinatal planning, and multidisciplinary management.

## 5. Conclusions

Although the identified pathogenic variant has been previously reported, this case illustrates the critical role of sequencing-based molecular diagnostics in enabling prenatal etiologic clarification, guiding perinatal management, and improving clinical preparedness in healthcare systems with limited access to genomic testing. By integrating molecular confirmation with prenatal imaging, an accurate etiologic diagnosis was achieved, thereby enabling early multidisciplinary intervention and targeted perinatal planning.

The findings are indicative of a more extensive systemic challenge in Bulgaria’s prenatal healthcare, as the country’s diagnostic precision, genetic counseling, and informed reproductive decision-making are all constrained by the limited availability of molecular genetic testing. Consequently, it is essential to expand insurance coverage for sequencing-based diagnostics to address this diagnostic gap and guarantee that clinical practice is in accordance with international genomic medicine standards.

The provision of comprehensive genetic counseling and coordinated specialist care is equally important, as they together facilitate early surveillance, complication prevention, and long-term outcome optimization for children who have been affected.

In general, this report underscores the importance of early molecular diagnosis in precision medicine for rare genetic disorders. It improves clinical preparedness, reduces irreversible morbidity, and resolves structural obstacles to equitable genomic healthcare. The diagnostic pathway described here may provide a clinically applicable framework for implementing prenatal molecular diagnosis of monogenic disorders in resource-constrained healthcare systems.

## Data Availability

The data presented in this study are available on request from the corresponding author. The data are not publicly available due to privacy and ethical restrictions involving sensitive participant information.
